# Influence of Drying Temperature and Harvesting Season on Phenolic Content and Antioxidant and Antiproliferative Activities of Olive (*Olea europaea*) Leaf Extracts

**DOI:** 10.3390/ijms24010054

**Published:** 2022-12-20

**Authors:** María Losada-Echeberría, Gustavo Naranjo, Dhafer Malouche, Amani Taamalli, Enrique Barrajón-Catalán, Vicente Micol

**Affiliations:** 1 Instituto de Investigación, Desarrollo e Innovación en Biotecnología Sanitaria de Elche (IDiBE), Universitas Miguel Hernández (UMH), 03202 Elche, Spain; 2 Department of Life Sciences, Universidad de las Fuerzas Armadas-ESPE, Sangolquí P.O. Box 171-5-231B, Quito, Ecuador; 3 Ecole Supérieure de la Statistique et de l’Analyse de l’Information, University of Carthage, 6 Rue des Métiers-Charguia II-B.P 675, Tunis 1080, Tunisia; 4 Laboratoire de Biotechnologie de l’Olivier, Centre de Biotechnologie de Borj-Cedria, B/P 901, Hammam-Lif 2050, Tunisia; 5 CIBEROBN (Physiopathology of Obesity and Nutrition CB12/03/30038), Carlos III Health Institute, 28029 Madrid, Spain

**Keywords:** antioxidant, antiproliferative, cancer, breast, colon, olive leaf extract, flavones

## Abstract

Interest in plant compounds has increased, given recent evidence regarding their role in human health due to their pleiotropic effects. For example, plant bioactive compounds present in food products, including polyphenols, are associated with preventive effects in various diseases, such as cancer or inflammation. Breast and colorectal cancers are among the most commonly diagnosed cancers globally. Although appreciable advances have been made in treatments, new therapeutic approaches are still needed. Thus, in this study, up to 28 olive leaf extracts were obtained during different seasons and using different drying temperatures. The influence of these conditions on total polyphenolic content (measured using Folin–Ciocalteu assays), antioxidant activity (using Trolox Equivalent Antioxidant Capacity and Ferric Reducing Ability of Plasma assays) and antiproliferative capacity (using 3-(4,5-dimethylthiazol-2-yl)-2,5-diphenyltetrazolium bromide, MTT assays) was tested in breast and colorectal cancer cells. Increased phenolic composition and antioxidant and antiproliferative capacity are noted in the extracts obtained from leaves harvested in autumn, followed by summer, spring and winter. Regarding drying conditions, although there is not a general trend, conditions using the highest temperatures lead to the optimal phenolic content and antioxidant and antiproliferative activities in most cases. These results confirm previously published studies and provide evidence in support of the influence of both harvesting and drying conditions on the biological activity of olive leaf extracts.

## 1. Introduction

Cancer is one of the major health problems in the population and is one of the leading causes of mortality worldwide. Specifically, breast cancer is the most common tumor in the world. In total, 1 in 8 women will have a breast tumor throughout her life, and 2.2 million new cases are diagnosed each year worldwide. In addition, breast cancer remains an unresolved disease as the annual rate of mortality is estimated to be approximately 700,000 deaths per year [1]. Colorectal cancer also ranks among the most common cancers. Specifically, colorectal cancer ranks fourth in incidence, with 1.9 million new cases diagnosed annually, and second in mortality, with approximately one million deaths per year.

As an alternative to conventional drugs, new treatments based on vegetal compounds are being studied. Some natural extracts and pure compounds have shown antiproliferative activity in breast cancer cell models [2,3,4]. The diversity of chemical structures, molecular targets and mechanisms of action is huge [5] but also promising, as it allows the development of new and significant studies that contribute to the continuous advancement of knowledge in this field.

Olive (*Olea europaea*) is probably one of the most famous sources of natural compounds with biological activity. Its extracts and compounds have been extensively reviewed [6,7,8,9], and most of its applications are related to cancer studies.

In this sense, olive leaf extracts have demonstrated activity against almost all of the most common cancers, including breast [10], hepatic [11] and colorectal cancer [12]. In addition, numerous studies have reported the antitumoral effects of other raw materials, such as oils and agri-food byproducts obtained from olive [13,14,15,16,17]. Pure compounds obtained from olives, olive products and olive-related industries have also been extensively studied [18,19,20,21]. Most of these studies are related to the antioxidant properties of these compounds [22,23]. However, anticancer research is also a common topic, especially breast cancer research [24,25,26]. The antiproliferative effect of olive extracts and compounds against breast cancer has been observed in numerous cellular [27,28,29] and animal models. A particular study described the use of olive leaf extracts in cancer. Specifically, the antiproliferative effect of an extract of olive leaf obtained using supercritical fluid extraction (SFE) was tested in cellular models of breast cancer (JIMT-1 cells) [30,31].

Numerous extractive techniques, including both classical (maceration, percolation, liquid–liquid extraction, among others) and novel techniques (such as SFE, ultrasounds or microwaves), have been used to obtain olive extracts and pure compounds [32,33,34,35,36,37]. Other studies have focused on pretreatments, such as drying [38,39,40]. The results are study-dependent, but the majority of the extractive techniques and pretreatments provided bioactive compounds with primary differences that centered on qualitative and quantitative composition as well as extraction yields. Another important factor to be studied is the influence of season on the yield and composition of the obtained products. This factor has been studied in other plants, such as seomcho [41], hypericum [42], asparagus [43] and oregano [44], as well as in olive [45,46,47].

The present work is the continuation of previous studies by our group focusing on the selection of the best olive leaf raw material for improved antiproliferative activity in cancer cell models [31], molecular characterization [48], identification of the intracellular metabolites present in the most active extract and their putative mechanisms of action [30]. In this new work, the influence of season and drying temperature is studied to identify the optimal harvesting and pretreatment conditions for further development.

## 2. Results

Up to 28 different extracts were obtained as described in the Methods section covering the four seasons and seven different drying conditions: fresh undried samples and samples dried at different temperatures (25, 40, 60, 80, 100 and 120 °C). The composition of each extract is fully described in [48].

### 2.1. Determination of Phenolic Content and Antioxidant Capacity

**Figure 1:**
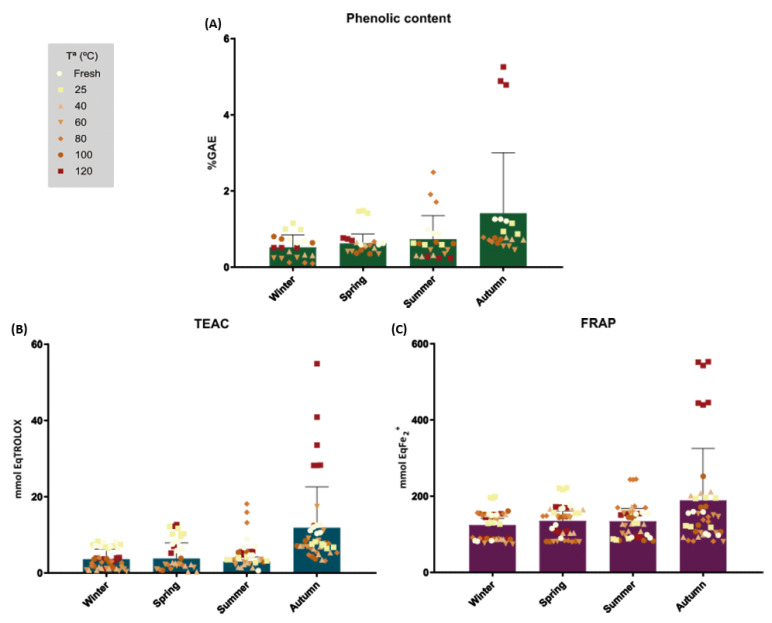
Phenolic content (**A**) and antioxidant capacity measured by TEAC (**B**) and FRAP (**C**) assays. Data are shown as a function of the collection season and drying temperature. (**A**) Data are expressed as the mean (n = 3) of the percentage of gallic acid equivalents per 100 g of each extract. (**B**) Data are expressed as the mean (n = 3) of mmol equivalents of Trolox per 100 g of each extract. (**C**) Data are expressed as the mean (n = 3) of mmol equivalents of Fe^2+^ per 100 g of each extract. Statistically significant differences were not noted among the samples.

The total polyphenolic content (TPC) was measured in all the samples as described in the Methods section. The highest phenolic content results obtained (Figure 1A and Table 1) were, individually, those of the AU120 extract with a mean %GAE of 4.98 ± 0.25, followed by SU80 at 2.04 ± 0.4 and SP25 at 1.45 ± 0.03. 

When grouped by season (Figure 1A), the best results were obtained for the autumn samples, followed by the summer, spring and winter extracts. When grouped by drying temperature, no clear influence of this parameter on TPC was noted.

The antioxidant activity was measured using two different methods, TEAC and FRAP, as described in the Methods section. The TEAC assay is a single-electron transfer-based method that has been used in a large variety of food samples (Huang et al., 2005), and Trolox served as a standard. The FRAP assay is also based on a single-electron transfer mechanism, but it is specifically used to determine the antioxidant capacity of biological samples.

The results are shown in Table 1 and Figure 1B,C. The highest antioxidant capacity results obtained for the TEAC assay were obtained from AU120 extract (autumn, 120 °C) with 32.75 ± 6.34 mmol Eq TROLOX followed by SU80 (summer, 80 °C) with 14.60 ± 1.94 mmol Eq TROLOX and SP25 (spring, 25 °C) with 11.99 ± 0.52 mmol Eq TROLOX (Figure 1B). For the FRAP assay, the same order was obtained, yielding values of 495.40 ± 2.17, 208 ± 1.71 and 188.14 ± 1.36 mmol EqFe^2+^ for AU120 (autumn, 120 °C), SU80 (summer, 80 °C) and SP25 (spring, 25 °C), respectively (Figure 1C).

Antioxidant assays rendered similar conclusions as TPC assays, showing that antioxidant activity was strongly related to TPC. A correlation was noted between TPC and both antioxidant tests, with coefficients of 0.94 for TEAC and 0.97 for FRAP. A similar relationship was observed between TEAC and the FRAP test (coefficient of 0.91). Regarding the influence of the season, autumn yielded the best results in the three tests, followed by summer, spring and winter. Overall, regarding the drying temperature, the results do not show a trend that allows for the determination of optimal conditions.

### 2.2. Determination of Antiproliferative Capacity

In order to determine the antiproliferative activity of the extracts, two breast cancer cell lines and a colorectal cancer cell line were selected based on their relevance as clinical models. MCF7 is a model estrogen receptor-dependent cell line that is representative of luminal A breast cancer. JIMT-1 is a model cell line of HER2-positive breast cancer that is resistant to Herceptin, and HCT-116 is a model colon cancer cell line with high migratory/invasive capacity.

**Figure 2:**
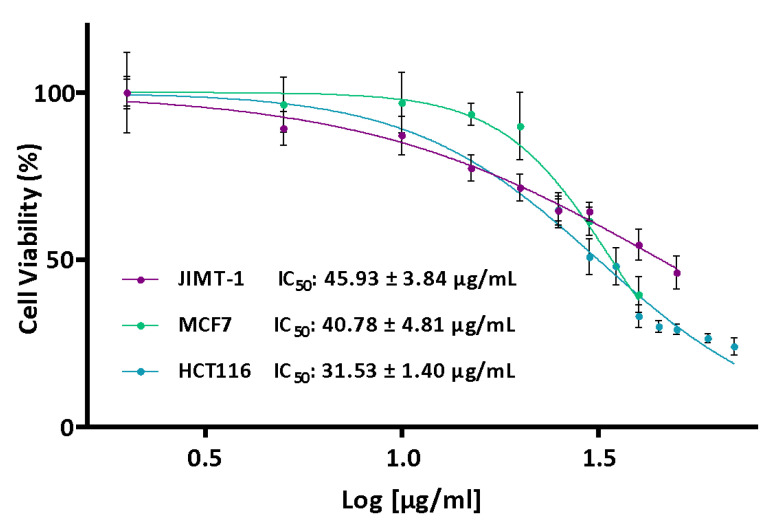
Effect of the SU80 extract on the MCF7 (Green) and JIMT-1 (Purple) breast cancer lines and HCT116 (Blue) colon cancer cell line. The cells were treated with different concentrations of the extract (0–70 μg/mL) for 24 h. Once the treatment was complete, cell viability was determined using the MTT assay. Values are presented as the percentage of cell viability (%, n = 6).

The different cell lines were treated using extract concentrations ranging between 0 and 70 µg/mL based on IC_50_ values obtained in previous studies [30]. However, in most cases, the IC_50_ values could not even be calculated due to the low antiproliferative activity of the extracts. The lowest IC_50_ values were obtained for the SU80 extract (summer, 80 °C) (Table 1). These values were 40.78 ± 4.81 µg/mL in MCF7 cells, 45.93 ± 3.84 µg/mL in JIMT-1 cells and 31.53 ± 1.40 µg/mL in HCT116 cells (Figure 2).

No correlations were observed among the IC_50_ values and total polyphenolic content or results for both antioxidant tests.

### 2.3. Statistical Search of Possible Candidates Responsible for the Activities of the Extracts

Based on previously published results [48] and the results described above, a statistical analysis was performed to determine the candidate compounds among all the compounds present in the extracts that are responsible for the antioxidant and antiproliferative activities of the extracts using a generalized linear model (GLM).

Statistical analysis was performed using R software. Variables with greater than 10 missing values were not analyzed to ensure the strength of the analysis; thus, MCF7 cell line results were not included in the analysis. The remaining variables, including bioactivities against colon (HCT116 cells) and breast cancer (JIMT-1 cells) and antioxidant activities, were used to estimate regressions using GLMs.

As shown in Table 2, oleuropein (isomer 1) was identified as demonstrating the greatest contribution to antioxidant activity based on the TEAC assay results, and the results are highly statistically significant (*p* < 0.001).

Regarding antioxidant activity measured using the FRAP method (expressed as mmolEqFe^2+^), GLM 2 revealed that vanillin was the candidate with the greatest contribution to antioxidant activity. This finding also exhibited high statistical significance (*p* < 0.001) (Table 3).

Regarding antiproliferative activity, acetoxypinoresinol and oleanolic acid were identified as the candidates with the greatest contributions in the GLM generated for colon cancer HCT116 cells, and the result was significant at *p <* 0.01 (Table 4). For breast cancer JIMT-1 cells, acetoxypinoresinol and ursolic acid were identified as the main compounds responsible for the antiproliferative activity in this cell line, and the results for both compounds were statistically significant (Table 5).

## 3. Discussion

The influence of harvesting time [49], drying [38,39,40,40,50,51] and extracting [32,33,34,35,36,37] conditions on the biological activity of olive leaf extracts and other vegetal matrices has been previously studied. These studies are difficult to compare because they employ different olive tree varieties, and most of these studies state that this is an important issue that strongly influences the polyphenolic content as well as the biological activities of the extracts [49]. However, despite this issue, all the studies confirm that harvesting during the summer yields an increased level of bioactive compounds as the plant uses these molecules as a defense against solar irradiation.

These previous results are also confirmed in our study, where summer and autumn harvesting render the best results for most of the tests developed. However, this study aims to go further and provide new insights into the identification of the compounds that influence these biological activities. Using qualitative and quantitative analyses of the composition of the different extracts as previously described [48], a statistical analysis was performed using a generalized linear model to determine the possible candidates for antioxidant and antiproliferative activity. Our results indicate that acetoxypinoresinol and ursolic acid contribute the most to the extract’s antiproliferative activity in JIMT-1 cells, whereas vanillin and oleuropein exhibit the greatest contributions to the extract’s antioxidant activity.

Previous results from our group [30] showed increased phenolic content and antioxidant and antiproliferative capacity, especially for the latter. Our previous results identified diosmetin, apigenin and luteolin as the extract components responsible for these biological activities [30]. However, the concentrations of these compounds are very low or nonexistent in the extracts assessed in the current study. Therefore, the observed effect must be exerted by compounds with less antiproliferative potential, namely, acetoxypinoresinol and ursolic acid, in this case. These results reaffirm the role of these compounds in the previously reported antiproliferative effects of the extract [30].

On the one hand, it is necessary to perform an extraction process aimed at increasing the content of these specific compounds to obtain the best results. For example, given the nonpolar nature of flavones, the extract could be separated into fractions based on polarity to increase the flavone concentration.

Furthermore, the low concentration or absence of these flavones may be attributed to the time of sample collection. In 2016 and 2017, the year of leaf collection, the Earth’s global surface temperatures were classified as the warmest since 1880 by the NOAA’s National Centers for Environmental Information [52,53], and it is known that the high temperatures, together with dry seasons can reduce polyphenol concentrations [54]. In addition, climate change may negatively influence the quality of the crops [55]. However, as mentioned above, the use of different varieties, species, extraction procedures and purification techniques introduces variability in the published results and makes any generic comparison difficult to achieve.

**Figure 3:**
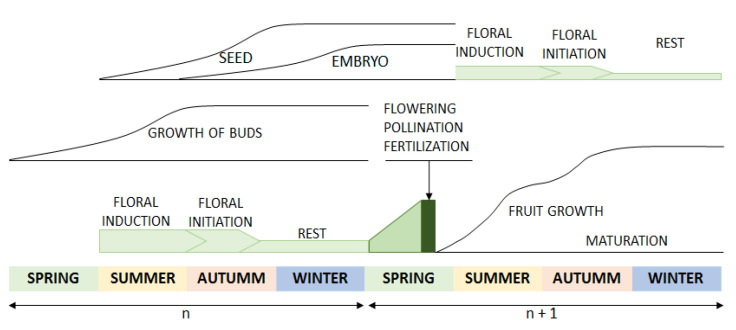
Vegetative and reproductive cycle of the olive tree.

Regarding the effect of the leaf harvesting season and the dry temperature, no statistical significance was obtained; nevertheless, a trend was observed regarding the effect of the seasonality of leaf collection. Leaves collected in winter are subject to the worst conditions. These conditions improve with the passing of the seasons until the best conditions are reached in autumn. Regarding the increase in drying temperature, the results do not show a trend that allows determining the optimal conditions. Other studies already published on the relationship among drying temperature, TPC and different biological activities have noted that higher drying temperatures have a positive influence on these parameters [50]. Our study also supports this conclusion; however, the results were not statistically significant, probably due to the intrinsic variability of the samples. These results can be explained if we consider the biennial cycle of the olive tree and its relationship to the progressive increase in phenolic content as previously described [56]. As seen in Figure 3, the olive tree is the least active, metabolically speaking, in the plant, in the winter months. The activities associated with the vegetative and reproductive cycle increase with the passing of the months until the maximum activity is reached in the autumn months. In addition, as a defense against UV radiation during the summer, phenolic compounds accumulate at greater levels during this time, thereby contributing to increased phenolic content in the autumn, as previously reported by other authors [57,58].

Finally, as this study focuses on in vitro tests, it has some limitations that should be addressed in future studies, mainly the influence of compounds’ bioavailability, metabolism and breast tissue distribution [59]. In this sense, the development of preclinical tests using animal models followed by human clinical trials is considered the main and most recommendable strategy.

## 4. Materials and Methods

### 4.1. Olive Leaf Extract Obtention

The whole collection of olive leaf samples was obtained from the olive cultivar ‘El Hor’ from the Center of Tunisia between January 2017 and November 2017 and covered the four seasons (winter, spring, summer and autumn). Samples were directly transferred to the laboratory, washed with distilled water and divided into groups depending on the treatment to be applied. The first aliquot of each group was immediately stored at −80 °C and labeled “fresh samples”. The remainder of the aliquots were dried either at room temperature (RT) (25 °C) or various temperatures of 40, 60, 80, 100 and 120 °C in a programmable mechanical convection oven (Binder Gmbh, Tuttlingen, Germany). Before processing using supercritical CO_2_ extraction (SFE), leaves were ground using an Ultra Centrifugal Mill ZM 200 (Retsch Gmbh, Haan, Germany). Further details about the extraction procedures, with particular attention given to SFE, are fully described in [48].

### 4.2. Folin–Ciocalteu Assay

For the study of phenolic compounds in the olive leaf extracts, 50 μL of Folin–Ciocalteu reagent was mixed with 10 μL of the sample, 100 μL of Na_2_CO_3_ 20% (*w*/*v*) and 840 μL of distilled water. Samples were incubated for 20 min at RT, and their absorbance at 700 nm was measured on a SPECTROstar Omega plate reader (BMG LabTech GmbH, Offenburg, Germany). The results were expressed as the mean gallic acid equivalents per 100 g dry weight.

### 4.3. TROLOX Equivalent Antioxidant Capacity (TEAC) Assay

First, ABTS^•+^ radical cations were generated by incubating the ABTS stock solution with potassium persulfate for 12–24 h at RT. For the study of phenolic compounds, the ABTS^•+^ solution was first diluted with distilled water to an absorbance of 0.700 at 734 nm and then processed as described in [60]. The final absorbance at 734 nm was measured using a SPECTROstar Omega plate reader. The results were expressed in millimoles equivalent of Trolox per 100 g of the compound.

### 4.4. Ferric Reducing Ability Power (FRAP) Assay

The FRAP method is a method used to estimate the reduction in a ferric−tripyridyltriazine (TPTZ) complex. For the study of phenolic compounds, 200 µL of freshly prepared FRAP reagent was mixed with 40 µL of distilled water or 40 µL of extract and incubated at 37 °C for 10 min. The FRAP reagent contained 2.5 mL of a 10 mM TPTZ solution, 2.5 mL of 20 mM ferric chloride and 25 mL of 300 mM sodium acetate. Absorbance was measured at 593 nm in a SPECTROstar Omega plate reader. A calibration curve was prepared with different concentrations of FeSO_4_ (0–300 µM). The results were expressed in millimoles of Fe^2+^ equivalents per 100 g of the compound.

### 4.5. Viability Assay

The cell lines used for antiproliferative assays included JIMT-1 (HER2-positive breast cancer) and MCF7 (luminal breast cancer) human breast carcinoma cells and HCT116 human colorectal cancer cells. All the cell lines were purchased from ATCC (Europe), except JIMT-1, which was purchased from the German Collection of Microorganisms and Cell Cultures (Braunschweig, Germany). Cells were plated in 96-well multiwell culture plates at a density of 7 × 10^3^ cells/well. After 24 h of incubation with the olive extracts (0–70 μg/mL), the antiproliferative effects of the extracts were determined using the MTT (3-(4,5-dimethylthiazol-2-yl)-2,5-diphenyltetrazolium bromide) cell proliferation assay. At the end of the treatment, a solution of the MTT reagent dissolved in a complete culture medium (250 μg/mL) was added and incubated for 3–5 h at 37 °C and 5% CO_2_. Then, the supernatant was removed from the wells, and 100 µL of DMSO was added per well to resuspend the formazan crystals. The plates were kept for 15 min under stirring at RT, and then the absorbance at 570 nm was measured in a SPECTROstar Omega plate reader using the absorbance at 620 nm as a reference. The results are expressed as the mean percentage of growth inhibition in 50% of the cell population (IC_50_ ± SD) relative to the control (n = 6).

### 4.6. Statistical Analysis of Composition and Activity

In order to determine the possible compounds responsible for the biological activities studied, statistical analysis of all the compounds present in the extracts and the biological activity data obtained was performed using R software (ver. 4.1.3). Her variables with greater than 10 missing values were discarded; thus, the antiproliferative activity against MCF7 cells was not included in the analysis. The resulting explanatory variables, including antioxidant activity, which was assessed based on both studied methods, and antiproliferative activity against JIMT-1 and HCT116, were used to estimate the regressions using a generalized linear model (GLM). Statistical significance for extraction conditions differences was tested through a Duncan test at a 5% confidence level using SPSS statistical package (Version 29.0.0.0 (241) for Windows, SPSS Inc., Chicago, IL, USA, 2003). 

## 5. Conclusions

The results obtained in this work show that olive leaf extracts obtained from leaves harvested in autumn, followed by summer, showed increased phenolic composition and antioxidant and antiproliferative capacity, probably as a result of increased solar irradiation. Additionally, these activities are positively influenced by drying temperature, as the higher the temperature, the better the results in most cases.

However, the method used to obtain olive leaf extracts does not offer sufficiently consistent reproducibility in terms of its composition and its biological activity, given the significant influence of the environmental conditions on the secondary metabolism mechanisms in this plant that produce high variability in the composition of its extracts.

Thus, it is recommended to work with combinations or mixtures of the pure compounds responsible for its biological activity, namely, diosmetin, apigenin and luteolin, to assess the antiproliferative effects of these compounds in cancer further.

## Figures and Tables

**Table 1:** Extraction conditions and results of phenolic content, antioxidant and antiproliferative capacity assays for the 28 olive leaf extracts. Statistical significance (*p* < 0.05) for each parameter (column) is indicated with letters, and different letters indicate statistically significant differences.

Extract	Season	Dry °C	%GAE	mmol EqTROLOX	mmol EqFe^2+^	IC_50_ MCF7	IC_50_ JIMT-1	IC_50_ HCT116
**WIF**	Winter	Fresh	0.69 ± 0.05 ^fgh^	7.71 ± 1.04 ^cdef^	117.13 ± 0.59 ^ab^	*^e^	67.84 ± 15.74 ^b^	65.24 ± 2.94 ^de^
**WI25**		25	1.05 ± 0.09 ^j^	7.68 ± 0.87 ^cdef^	162.71 ± 0.05 ^abc^	*^e^	* ^d^	139.10 ± 26.15 ^i^
**WI40**		40	0.34 ± 0.05 ^bc^	1.48 ± 0.13 ^a^	119.47 ± 0.08 ^ab^	*^e^	* ^d^	*^j^
**WI60**		60	0.24 ± 0.01 ^ab^	1.31 ± 0.14 ^a^	108.02 ± 0.31 ^a^	*^e^	* ^d^	*^j^
**WI80**		80	0.11 ± 0.01 ^a^	3.25 ± 0.54 ^abc^	121.89 ± 1.38 ^ab^	*^e^	* ^d^	82.69 ± 2.60 ^g^
**WI100**		100	0.73 ± 0.08 ^hg^	2.88 ± 0.77 ^abc^	121.74 ± 2.19 ^ab^	*^e^	* ^d^	*^j^
**WI120**		120	0.51 ± 0.00 ^cdef^	3.65 ± 0.67 ^abc^	119.55 ± 3.34 ^ab^	*^e^	* ^d^	*^j^
**SPF**	Spring	Fresh	0.60 ± 0.02 ^efgh^	7.54 ± 1.45 ^cdef^	145.31 ± 1.57 ^abc^	56.58 ± 8.59 ^c^	71.75 ± 22.77 ^b^	47.50 ± 1.64 ^c^
**SP25**		25	1.45 ± 0.03 ^l^	11.99 ± 0.52 ^f^	188.14 ± 1.36 ^cd^	53.43 ± 5.20 ^bc^	* ^d^	94.97 ± 2.11 ^hg^
**SP40**		40	0.58 ± 0.08 ^defgh^	2.06 ± 0.99 ^ab^	134.69 ± 0.21 ^ab^	*^e^	* ^d^	*^j^
**SP60**		60	0.39 ± 0.04 ^bcd^	2.25 ± 0.23 ^ab^	113.52 ± 1.16 ^ab^	*^e^	* ^d^	64.36 ± 1.92 ^de^
**SP80**		80	0.59 ± 0.07 ^efgh^	1.99 ± 0.12 ^a^	115.24 ± 1.18 ^ab^	*^e^	* ^d^	*^j^
**SP100**		100	0.39 ± 0.05 ^bcd^	2.07 ± 0.70 ^ab^	113.65 ± 0.37 ^ab^	*^e^	* ^d^	98.58 ± 2.19 ^g^
**SP120**		120	0.74 ± 0.03 ^hg^	9.71 ± 0.28 ^def^	139.02 ± 1.18 ^ab^	*^e^	* ^d^	85.16 ± 1.88 ^g^
**SUF**	Summer	Fresh	0.91 ± 0.07 ^gj^	6.51 ± 2.17 ^bcde^	125.31 ± 2.01 ^ab^	*^e^	* ^d^	*
**SU25**		25	0.61 ± 0.02 ^efgh^	3.94 ± 0.73 ^abc^	108.15 ± 0.90 ^a^	*^e^	* ^d^	86.69 ± 1.36 ^f^
**SU40**		40	0.30 ± 0.02 ^b^	2.51 ± 1.12 ^abc^	108.27 ± 0.10 ^a^	*^e^	* ^d^	*^j^
**SU60**		60	0.42 ± 0.06 ^bcde^	3.20 ± 0.53 ^abc^	127.87 ± 3.04 ^ab^	*^e^	* ^d^	83.10 ± 2.19 ^g^
**SU80**		80	2.04 ± 0.40 ^m^	14.88 ± 1.94 ^f^	208.00 ± 1.71 ^d^	40.78 ± 4.8 ^a^	45.93 ± 3.84 ^a^	31.53 ± 1.40 ^a^
**SU100**		100	0.63 ± 0.02 ^efgh^	4.72 ± 1.80 ^abc^	114.80 ± 2.07 ^ab^	*^e^	* ^d^	67.83 ± 1.88 ^e^
**SU120**		120	0.25 ± 0.02 ^ab^	5.78 ± 0.15 ^abcd^	123.07 ± 0.44 ^ab^	*^e^	* ^d^	61.34 ± 2.17 ^d^
**AUF**	Autumn	Fresh	1.24 ± 0.03 ^k^	11.98 ± 1.40 ^f^	128.68 ± 0.42 ^ab^	60.39 ± 6.47 ^c^	69.55 ± 19.68 ^b^	40.06 ± 1.23 ^b^
**AU25**		25	0.99 ± 0.15 ^j^	7.74 ± 0.90 ^def^	158.08 ± 0.68 ^abc^	*^e^	* ^d^	97.70 ± 2.36 ^g^
**AU40**		40	0.74 ± 0.03 ^hg^	6.06 ± 1.78 ^abcd^	151.21 ± 0.90 ^abc^	91.0 ± 28.55 ^d^	66.02 ± 9.11 ^b^	92.72 ± 5.33 ^h^
**AU60**		60	0.52 ± 0.06 ^degh^	9.20 ± 1.38 ^def^	127.49 ± 2.86 ^ab^	46.23 ± 2.84 ^ab^	104.80 ± 21 ^c^	45.09 ± 0.09 ^c^
**AU80**		80	0.72 ± 0.06 ^h^	6.56 ± 1.21 ^bcde^	110.98 ± 3.8 ^ab^	*^e^	* ^d^	64.58 ± 3.04 ^de^
**AU100**		100	0.71 ± 0.07 ^gh^	9.41 ± 2.33 ^def^	139.58 ± 0.81 ^abc^	*^e^	107.09 ± 38.99 ^c^	77.10 ± 3.0 ^f^
**AU120**		120	4.98 ± 0.25 ^n^	33.17 ± 6.53 ^g^	495.40 ± 2.17 ^e^	*^e^	* ^d^	37.17 ± 3.00 ^b^

* IC_50_ > 100 μg/mL.

**Table 2:** GLM 1 summary (antioxidant capacity, TEAC).

Deviance Residuals					
Min	1Q	Median		3Q	Max
−4.407	−2.439	−0.835		1.476	10.217
**Coefficients**					
	**Estimate**	**SEM**	**T Value**	**Pr (>|t|)**	**Significance**
(Intercept)	4.52269	0.65513	6.904	1.67 × 10^−7^	*p* < 0.001
Oleuropein isomer 1	0.13492	0.01532	8.805	1.48 × 10^−9^	*p* < 0.001

**Table 3:** GLM 2 Summary (antioxidant capacity, FRAP).

Deviance Residuals					
Min	1Q	Median		3Q	Max
−66.299	−12.195	−5.510		9.605	83.146
**Coefficients**					
	**Estimate**	**SEM**	**T Value**	**Pr (>|t|)**	**Significance**
(Intercept)	125.44683	6.15193	20.391	<2 × 10^−16^	*p* < 0.001
Vanillin	−0.85119	0.14673	−5.801	3.58 × 10^−6^	*p* < 0.001

**Table 4:** GLM 3 summary (antiproliferative capacity, JIMT1).

Deviance Residuals					
Min	1Q	Median		3Q	Max
−164.10	−88.79	−20.14		27.46	348.75
**Coefficients**					
	**Estimate**	**SEM**	**T Value**	**Pr (>|t|)**	**Significance**
(Intercept)	214.510	31.375	6.837	2.41 × 10^−7^	*p* < 0.001
Acetoxypinoresinol	−35.780	6.890	−5.193	1.81 × 10^−5^	*p* < 0.001
Ursolic acid	26.582	5.148	5.164	1.96 × 10^−5^	*p* < 0.001

**Table 5:** GLM 4 summary (antiproliferative capacity, HCT116).

Deviance Residuals					
Min	1Q	Median		3Q	Max
−50.761	−5.184	−0.502		10.423	51.635
**Coefficients**					
	**Estimate**	**SEM**	**T Value**	**Pr (>|t|)**	**Significance**
(Intercept)	90.255	4.562	19.783	<2 × 10^−16^	*p* < 0.001
Acetoxypinoresinol	−2.315	1.193	−1.940	6.28 × 10^−3^	*p* < 0.05
Oleanolic acid	−3.207	1.022	−3.138	4.08 × 10^−3^	*p* < 0.01

## Data Availability

Not applicable.
